# Role of Apolipoprotein A1 in PPAR Signaling Pathway for Nonalcoholic Fatty Liver Disease

**DOI:** 10.1155/2022/4709300

**Published:** 2022-02-18

**Authors:** Changxi Chen, Hongliang Li, Jian Song, Cheng Zhang, Mengting Li, Yushan Mao, Aiming Liu, Juan Du

**Affiliations:** ^1^Department of Gastroenterology, Affiliated People's Hospital of Ningbo University, Ningbo, Zhejiang Province 315040, China; ^2^Department of Endocrinology, Affiliated Hospital of Medical College of Ningbo University, Ningbo, Zhejiang Province 315020, China; ^3^School of Medicine, Ningbo University, Ningbo 315211, China; ^4^Department of Gastroenterology, Refine-Chemical Hospital of Zhenhai District, Ningbo, Zhejiang Province 315207, China

## Abstract

Peroxisome proliferator-activated receptors (PPARs) have been suggested to play crucial roles in the pathology of NAFLD with a vague understanding of the underlying mechanism. Here, we integrated large-scale literature data and clinical data to explore the potential role of the PPAR-APOA1 signaling pathway in the pathology of NAFLD. First, the signaling pathway connecting PPARs, APOA1, and NAFLD was constructed. Then, we employed clinical data to explore the association between APOA1 levels and NAFLD. In addition, we built the APOA1-driven pathway analysis to explore the potential mechanism of the APOA1-NAFLD association. Pathway analysis showed that APOA1 serves as a hubprotein connecting PPARs and NAFLD through a beneficial modulation of 16 out of 21 NAFLD upstream regulators. Each relationship within the composed pathway was supported by results from multiple previous studies. Clinical data analysis showed that an increase of APOA1 level was associated with a significantly decreased NAFLD prevalence (*χ*^2^ = 292.109; *P* < 0.001). When other confounding factors were adjusted, serum APOA1 level was shown as an independent risk factor for the prevalence of NAFLD (*P* value<.0001; OR = 0.562). Our results suggested that the three PPARs (PPARA, PPARD, and PPARG) might promote the expression and molecular transportation of APOA1 to form a PPAR-APOA1 signaling pathway that demonstrated a beneficial role in the pathogenesis of NAFLD.

## 1. Introduction

Nonalcoholic fatty liver disease (NAFLD) is characterized by an excessive fat build-up in the liver without a clear cause, such as alcohol use [1, 2]. NAFLD is also known as a metabolic dysfunction-associated fatty liver disease that has two subtypes, nonalcoholic fatty liver (NAFL) and nonalcoholic steatohepatitis (NASH). Compared with NAFL, NASH is more dangerous with liver inflammation [3]. NAFLD, especially when it progresses to NASH, may eventually lead to complications such as liver failure, liver cancer, cirrhosis, or cardiovascular disease [4].

Peroxisome proliferator-activated receptors have been suggested to play crucial roles in the pathology of NAFLD [5, 6]. Specifically, peroxisome proliferator-activated receptor *δ* (PPARD) and peroxisome proliferator-activated receptor *α* (PPARA) have been suggested as therapeutic targets to alleviate NAFLD [5, 7]. Peroxisome proliferator-activated receptor *γ* (PPARG) has also been shown essential to protect against nonalcoholic steatohepatitis [6]. However, the underlying mechanism regarding the roles of PPARs in NAFLD remains vague and controversial [8, 9].

As the major component of high-density lipoprotein (HDL) particles, apolipoprotein A1 (APOA1) is a protein encoded by the APOA1 gene to have a specific role in lipid metabolism [10, 11]. The APOA1 gene is located on the 11th chromosome, with its specific location being 11q23-q24. APOA1 is the major protein component of HDL particles in plasma; it enables efflux of fat molecules by accepting fats from within cells and transport elsewhere, including back to LDL particles or the liver for excretion. APOA1 helps clear fats from white blood cells within artery walls to keep from becoming fat overloaded, transforming into foam cells that contribute to progressive atheroma. It has been shown that APOA1 levels were significantly decreased in NAFLD patients [12], which increase the risk of NAFLD developing [13]. Ren et al.'s study found that both APOA1 and APOB and TC/HDL-C had the predictive value of NAFLD [14]. On the other hand, modulation of APOA1 activity leads to a beneficial effect on NASH [15].

All three PPARs (PPARA, PPARD, and PPARG) were implicated as promoters to increase APOA1 secretion and expression from the liver [16–18], which may partially decode the role of PPARs in NAFLD. Here, we employed large-scale literature-based pathway analysis and clinical data analysis to explore the role of the PPAR-APOA1 signaling pathway in NAFLD, which may add new insights into the understanding of the NAFLD treatment.

## 2. Materials and Methods

The rest of this study was organized as follows. First, we conducted a literature-based pathway analysis to study the PPAR-APOA1 signaling pathway and its role in the pathology of NAFLD. Second, we used large-scale clinical data to study the association between APOA1 levels and NAFLD. In addition, we constructed the genetic and small molecule pathways to explore the potential mechanism of APOA1-NAFLD association.

### 2.1. PPAR-APOA1 Signaling Pathway for NAFLD

**Figure 1:**
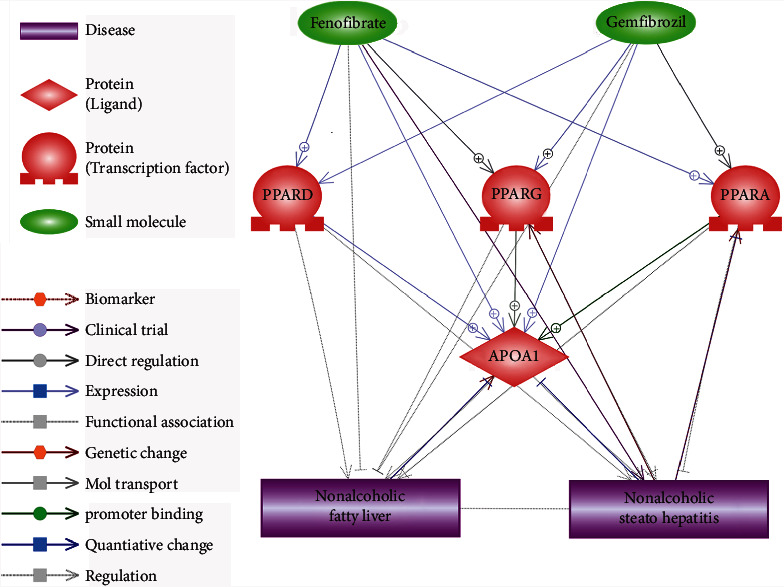
PPAR-APOA1 signaling pathway regulating nonalcoholic fatty liver disease.

**Figure 2:**
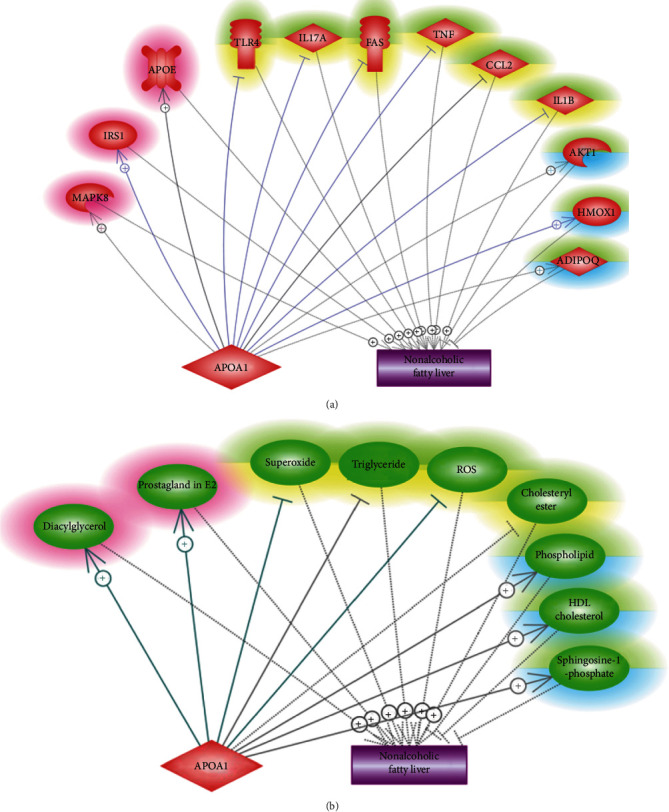
Molecule pathways that decode the APOA1-NAFLD relationship. (a) The APOA1-driven genetic pathway. (b) The APOA1-driven small molecular pathway.

Assisted by Elsevier Pathway Studio (http://www.pathwaystudio.com) knowledge database, we constructed the PPAR-APOA1 signaling pathway to explore the connection between PPARs, APOA1, and NAFLD. The entities within the network included the three PPARs (PPARA, PPARD, and PPARG), the protein APOA1, the diseases (nonalcoholic fatty liver and nonalcoholic steatohepatitis), and the two potential drugs for the treatment of NAFLD (fenofibrate and gemfibrozil). The relationships between the entities were identified by using the network building module (https://supportcontent.elsevier.com/Support%20Hub/Pathway%20Studio/Guide%20to%20Building%20Pathways%20in%20ChemEffect%20and%20DiseaseFX%20with%20Pathway%20Studio%20Web.pdf). The relationship between the entities within the signaling pathway was supported by at least three references. Then, a manual quality control process was conducted to ensure the reliability of the relationships. A reference list was provided in Supplementary Material 1, which is a two-worksheet excel file described as follows. The worksheet “Ref for PPARs-APOA1 pathway” contains reference information supporting the PPAR-APOA1 pathway (Figure 1), including the type of the relationship, supporting references, and related sentences from the references where the relationship has been identified. The worksheet “Ref for APOA1 Molecule pathway” contains reference information supporting the genetic and molecule pathways (Figure 2), including the type of the relationship, supporting references, and related sentences from the references where the relationship has been identified.

### 2.2. APOA1 Levels in NAFLD Clinical Data

#### 2.2.1. Subjects in the Clinical Data

Clinical data were collected from inservice and retired employees who underwent physical examinations in the health examination center of Zhenhai Lianhua Hospital from March to November 2013. There were 14,320 questionnaires and physical examination forms with 257 undetected patients excluded, resulting in a total of 14,063 cases for physical examination; out of these 14,063 subjects, 1,240 subjects that met the exclusion criteria were removed from the data collection. Finally, a total of 12,823 subjects were included (8,479 males and 4,344 females). The average age was (47.2 ± 15.1) years. Of these 12,823 subjects, 3147 (24.5%) fulfilled the diagnostic criteria of NAFLD, and the prevalence in men and women was 28.8% and 16.3%, respectively. More information regarding the clinical data and related data process is available in Supplementary Material 2.

#### 2.2.2. APOA1 Levels and Their Association with NAFLD

Fasting venous blood from the cubital vein and centrifuged was used to prepare the serum for biochemical analysis. The APOA1 levels were studied using the AU640 fully automatic biochemical analyzer (Olympus, Kobe, Japan). To explore the relationship between APOA1 levels and the prevalence of NAFLD, the 12,823 subjects were separated into four different groups according to their serum APOA1 levels, which were described as follows. (1) Q1 group: APOA1 level ≤ 1.18 g/L; (2) Q2 group: APOA1 level was between 1.19 and 1.32 g/L; (2) Q3 group: APOA1 level was between 1.33 and 1.55 g/L; and (2) Q4 group: APOA1 level ≥ 1.56 g/L in the Q4 group. A chi-square test was used to test the prevalence of NAFLD under different APOA1 levels. Multivariate logistic regression was used to analyze corisk factors of NAFLD besides APOA1 levels.

#### 2.2.3. APOA1-Driven Signaling Pathway for NAFLD

Assisted by the literature-based Pathway Studio (http://www.pathwaystudio.com) knowledge database, we identified common genes and small molecules that were downstream targets of APOA1 and upstream regulators of NAFLD. These relation data were then used to compose the APOA1 seeded pathways, which was tentative to explore the possible mechanisms underlying the role of APOA1 in the pathology of NAFLD. A manual quality control process was conducted to ensure the reliability of the relationships and their polarities. A reference list was provided in Supplementary Material 1.

## 3. Result

### 3.1. PPAR-APOA1 Signaling Pathway for NAFLD

Literature-based pathway analysis showed that PPARs promote the synthesis and secretion of APOA1 in hepatocytes, as shown in Figure 1. The PPARD➔APOA1 relationship was supported by six independent studies; the PPARG➔APOA1 relationship was supported by 11 independent studies, and over 30 studies supported the PPARA➔APOA1 relationship. Regarding the association between APOA1 and NAFLD, on the one hand, two studies showed that modulation of APOA1 activity might have a beneficial effect on NASH, and the deficiency of APOA1 facilitates hepatic diet-induced deposition of triglycerides and NAFLD development in mice. On the other hand, multiple studies showed that serum APOA1 levels were significantly decreased in NAFLD patients. These previous studies support APOA1 as a vital hub protein within the PPAR pathway to play a beneficial role in the development of NAFLD.

Moreover, two drugs (fenofibrate and gemfibrozil) employed in NAFLD treatment were identified as promoters for all three PPARs, which also increase APOA1 mRNA in human liver biopsies as well as APOA1 plasma concentration. These studies extended the PPAR-APOA1 signaling pathway and provided further support for its role in NAFLD pathology. In total, over 1,200 references support the relationships presented in Figure 1, which is available at the worksheet of “Ref for PPARs-APOA1 pathway” in Supplementary Material 1.

### 3.2. Clinical Study Supporting APOA1–NAFLD Relationship

Due to the limited support from previous studies regarding the role of serum APOA1 in NAFLD, in this study, we employed large-scale clinical data to test the association between the variation of APOA1 levels and the prevalence of NAFLD. Our results indicated that the prevalence of NAFLD could decrease with elevated APOA1 levels, and that low serum APOA1 levels might be an independent risk factor for the prevalence of NAFLD.

#### 3.2.1. Prevalence of NAFLD

NAFLD prevalence in Q1, Q2, Q3, and Q4 groups was 32.58%, 28.21%, 21.55%, and 15.58%, respectively. With the increase of APOA1 concentration, the prevalence of NAFLD was significantly decreased (*χ*^2^ = 292.109, *P* < 0.001), as shown in Table 1.

#### 3.2.2. Multivariate Logistic Regression Analysis of Risk Factors of NAFLD

We explored the possibility of the decreased APOA1 levels as a risk for NAFLD incidence with/without adjusting other confounding factors and presented the results in Table 2. In the unadjusted model (model 1), compared with individuals in baseline serum APOA1 levels in group 1, the odds ratios (ORs) and 95% confidence intervals (CI) for NAFLD prevalence were 0.814 (0.731-.905), 0.568 (0.508-0.636), and 0.382 (0.339-0.431) for individuals in group 2, group 3, and group 4, respectively. With the increase of APOA1 levels (group 1 to group 4), the ORs for NAFLD prevalence were decreased, indicating the beneficial role of increased APOA1 levels in NAFLD.

When other risk factors were adjusted (model 2 and model 3), the ORs were increased, suggesting APOA1 as an independent risk factor for NAFLD. Especially in the group where APOA1 levels were high (group 3 and group 4), the influence of APOA1 levels was statistically significant in all models with/without adjusting other confounding factors. These results indicated that the increased serum APOA1 levels could be more critical than the deficiency of serum APOA1 in their influence on NAFLD development. Our results supported the APOA1–NAFLD relationship within the PPAR-APOA1 signaling pathway constructed in Figure 1.

### 3.3. APOA1 Seeded Signaling Pathway for NAFLD

Both literature-based pathway analysis and our clinical data suggested that increased APOA1 levels might play a beneficial role in NAFLD. To understand the underlying mechanisms, we conducted a literature-based molecule pathway analysis to identify downstream targets of APOA1 that were also upstream regulators of NAFLD, based on which APOA1-driven signaling pathways influencing NAFLD development were constructed and presented in Figure 2.

Overall, APOA1 beneficially regulated 16 out of 21 NAFLD regulators, highlighted in green-yellow or green-blue in Figures 2(a) and 2(b)). Specifically, APOA1 inhibited 10 NAFLD promoters (highlighted by green-yellow) and activated six NAFLD inhibitors (highlighted by green-blue), which might partially explain the beneficial role of APOA1 in the pathology of NAFLD. However, we also noted that increased APOA1 might promote five NAFLD promoters (highlighted in red), adding complexity to the APOA1-NAFLD relationship. The supporting references and the corresponding descriptive sentences for the relationships presented in Figures 2(a) and 2(b)) were provided in the worksheet of “Ref for APOA1 Molecule pathway” of Supplementary Material 1.

## 4. Discussion

All three peroxisome proliferator-activated receptors (PPARA, PPARD, and PPARG) attenuate NAFLD and thus were suggested as therapeutic targets for the treatment of NAFLD [5–7]. Results from a recent phase 2b clinical trial study showed that the pan-PPAR agonist lanifibranor could modulate key metabolic, inflammatory, and fibrogenic pathways in the pathogenesis of NASH, indicating the beneficial effect of the activation of PPARs on NAFLD [17]. However, the mechanism regarding PPAR-NAFLD regulation remains vague [8, 9]. Results from this study showed that APOA1 could be a hubprotein within the PPAR signaling pathway regulating the pathologic development of NAFLD.

Multiple previous studies supported the positive regulation of PPARs on APOA1. For instance, Gervois et al. showed that the PPARA activation could induce hepatic APOA1 and APOA2 expression in humans and lead to increased plasma HDL cholesterol [18]. Singh et al.'s study suggested that hepatic APOA1 and APOA2 expression could also be increased by the activation of PPARG [19]. In addition, PPARD has been reported to increase APOA1 and high-density lipoprotein synthesis through the activation of the ATP-binding cassette transporter 1 (ABCA1) gene [20]. These studies supported that APOA1 could be a common downstream target for all three PPARs.

Moreover, multiple studies supported the association between decreased levels of APOA1 and the prevalence of NAFLD [13, 20], and one study suggested a beneficial effect of increased APOA1 levels on NAFLD [15]. Results from our clinical data analysis confirmed that elevated serum APOA1 levels might function as an independent protective factor for the development of NAFLD (Tables 1 and 2). Taken together, these results suggested that increased APOA1 levels within the PPAR-APOA1 pathway could exert a beneficial role in protecting against the development of NAFLD.

Interestingly, literature-based pathway analysis also showed that PPARs (PPARA, PPARD, and PPARG), as well as APOA1, were downstream targets of two drugs (fenofibrate and gemfibrozil) that have the potential to be used in the treatment of NAFLD [21, 22], as shown in Figure 1. These results extended the PPAR-APOA1 signaling pathway and added support to its role in NAFLD.

As the mechanism of the APOA1-NAFLD relationship was largely unknown, we conducted another literature-based molecule pathway analysis. Our results showed that APOA1 could modulate 16 out of 21 NAFLD regulators in favor of a protective role against NAFLD development (Figure 2). For example, APOA1 could decrease the mRNA expression and production of IL1B [23], which plays a vital role in the pathologic development of NAFLD [24]. This built an APOA1--|IL1B--+ > NAFLD pathway. Another example was the APOA1--+ > ADIPOQ--|NAFLD pathway. APOA1 mimetic has been shown to induce the expression of ADIPOQ [25], which inhibits NAFLD by reducing fat content and promoting fatty acid oxidation [26]. For more of these pathways and their supporting references, please refer to Figure 2 and the worksheet “Ref for APOA1 Molecule pathway” of Supplementary Material 1. These pathways might add new insights into the understanding of the role of APOA1 in the pathogenesis of NAFLD.

This study has one limitation as follows. The PPAR-APOA1 pathway was built mainly based on previous studies and partially on clinical data. Due to the complexity of the relationship between PPARs and NAFLD, the composed pathway in this study should be tested in a biology experiment.

## 5. Conclusions

This study integrated literature-based pathway analysis and clinical data analysis to study the PPAR-APOA1 signaling pathway and their role in the pathologic development of NAFLD. Our results showed that the three PPARs (PPARA, PPARD, and PPARG) might promote the expression and molecular transportation of APOA1, which mainly plays a beneficial role in the development of NAFLD. Our results may add new insights into the understanding of the role PPARs play in NAFLD.

## Figures and Tables

**Table 1:** The prevalence of NAFLD decreased with the increase of APOA1 level.

APOA1 levels	Total patients	Patients with NAFLD	Prevalence rate	*χ*2 value	*P* value
Q1	3300	1075	32.58%	292.109	<0.001
Q2	3165	893	28.21%		
Q3	3156	680	21.55%		
Q4	3202	499	15.58%		

**Table 2:** MLR results of risk factors of NAFLD according to serum APOA1 quintiles.

APOA1	*n*	Model 1		Model 2		Model 3	
Quartile		*P* value	OR (95% CI)	*P* value	OR(95% CI)	*P* value	OR (95% CI)
Q1	3300		1		1		1
Q2	3165	<0.000	0.814 (0.731-.905)	0.806	0.984 (0.869-1.115)	0.479	1.055 (0.913-.220)
Q3	3156	<0.000	0.568 (0.508-0.636)	0.002	0.812 (0.711-0.927)	0.005	0.790 (0.670-.932)
Q4	3202	<0.000	0.382 (0.339-0.431)	<.0001	0.625 (0.539-0.724)	<.0001	0.562 (0.452-.699)

Note: model 1 does not adjust for confounding factors; model 2 adjusted age, gender, and body mass index; model 3 adjusted age, sex, body mass index, waist circumference, uric acid, systolic blood pressure, diastolic blood pressure, total cholesterol, triacylglycerol, low-density lipoprotein cholesterol, high-density lipoprotein cholesterol, fasting blood glucose, alanine aminotransferase, aspartate aminotransferase, and glutamyltranspeptidase.

## Data Availability

All data generated or analyzed during this study are available upon request.

## Supplementary Materials

Supplementary 1 Supplementary Material 1 is a two-worksheet excel file described as follows. The worksheet “Ref for PPARs-APOA1 pathway” contains reference information supporting the PPAR-APOA1 pathway (Figure 1), including the type of the relationship, supporting references, and related sentences from the references where the relationship has been identified. The worksheet “Ref for APOA1 Molecule pathway” contains reference information supporting the genetic and molecule pathways (Figure 2), including the type of the relationship, supporting references, and related sentences from the references where the relationship has been identified.

Supplementary 2 Supplementary Material 2 presents more detailed information of the clinical data and related data process, including participant's exclusion rules, general information collection process, blood biochemical index tests process, hepatic ultrasound examination process, statistical analysis method description, and background clinical data of the patients.
