# Supplementary material for: Role of Apolipoprotein A1 in PPAR Signaling Pathway for Nonalcoholic Fatty Liver Disease
Source: PPAR Res. 2022 Feb 18;2022:4709300. doi: 10.1155/2022/4709300 (PMC8886744; doi:10.1155/2022/4709300)
Supplement: Supplementary 1 — Supplementary Material 1 is a two-worksheet excel file described as follows. The worksheet “Ref for PPARs-APOA1 pathway” contains reference information supporting the PPAR-APOA1 pathway (Figure 1), including the type of the relationship, supporting references, and related sentences from the references where the relationship has been identified. The worksheet “Ref for APOA1 Molecule pathway” contains reference information supporting the genetic and molecule pathways (Figure 2), including the type of the relationship, supporting references, and related sentences from the references where the relationship has been identified. [file 4709300.f1.docx]

Supplementary material 1

The Supplementary material 1 is a two-worksheet excel file that contains described as follows.

1. **Ref for PPARs-APOA1 pathway**: Reference information regarding PPARs-APOA1 Pathway was presented, including the type of the relationship, supporting references, and related sentences from the references where the relationship has been identified.
2. **Ref for APOA1 Molecule pathway**: Reference information regarding genetic and molecule pathways was presented, including the type of the relationship, supporting references, and related sentences from the references where the relationship has been identified.

The excel file is online available at: [www.gousinfo.com/database/Data_Genetic/NAFLD_APOA1_PPAR.xlsx](http://www.gousinfo.com/database/Data_Genetic/NAFLD_APOA1_PPAR.xlsx%20)
