# Supplementary material for: Role of Apolipoprotein A1 in PPAR Signaling Pathway for Nonalcoholic Fatty Liver Disease
Source: PPAR Res. 2022 Feb 18;2022:4709300. doi: 10.1155/2022/4709300 (PMC8886744; doi:10.1155/2022/4709300)
Supplement: Supplementary 2 — Supplementary Material 2 presents more detailed information of the clinical data and related data process, including participant's exclusion rules, general information collection process, blood biochemical index tests process, hepatic ultrasound examination process, statistical analysis method description, and background clinical data of the patients. [file 4709300.f2.docx]

**Supplementary material 2: Details regarding clinical data**

**Participant’s exclusion rules:**

The following participants were excluded: (1) those who had fatty liver at baseline; (2) those with excessive of alcohol consumption (>140g per week for men and >70 g per week for women); (3) those who had viral hepatitis, Wilson's disease, autoimmune liver disease, drug-induced liver disease, etc.; (4) those with uncompleted data. The study was approved by the Ethics Committee of our institution [Approval number: Zhenhai Lianhua Hospital (2012) No. 2], and all the participants signed the informed consent form.

**General information collection**

After a short-term training for investigators, the questionnaire survey and relevant examinations for the people were uniformly conducted. The content of the questionnaire included the drinking and smoking history, history of liver diseases and whether they are taking hepatoprotective drugs.clinical examinations were carried following standard process. Height, weight, and waist circumference were measured by standard methods in an early morning fasting state. The midpoint of the lower edge of the ilium and rib was used for level measurement. Patients took a 5-minute break in a quiet setting before systolic and diastolic pressure was measured.

**Blood biochemical index tests**

A 10 ml volume of fasting venous blood was drawn from the cubital vein and centrifuged to prepare the serum was which was subsequently used for biochemical analysis. ApoA1 levels was tested using the AU640 fully automatic biochemical analyzer (Olympus, Kobe, Japan). The analysis was conducted by experimental methodological tests.

**Hepatic ultrasound examination**

After phlebotomy, the subjects underwent hepatic ultrasound examination after fasting plasma glucose. Trained ultrasonographists carried out ultrasonic diagnostic using the same ultrasound machine model to perform the scans and issue a report. The ultrasonographists did not know about the study or the history of the patients. The diagnosis of NAFLD was based on the clinical diagnosis standard of the Guidelines for the Diagnosis and Treatment of NAFLD (Revised Edition 2010) from the Hepatology Branch of the Chinese Medical Association.

**Statistical analysis**

Continuous variables were expressed as the mean ± standard deviation or median ± quartile interval. Continuous variables were compared using the Student’s t-test or Mann-Whitney U-test. Categorical data were compared using the chi square test. Univariate and multivariate logistic regression analysis (backward: Wald; cut off for entry: 0.05, for removal: 0.10) was applied to assess the risk factors for NAFLD. All statistical analyses were performed using SPSS 22.0 software for Windows (SPSS Inc., Chicago, IL). A P<0.05 was considered statistically significant.

**Relationship between serum apolipoprotein A1 level and metabolic syndrome and related indexes:**

The characteristics of 12823 participants who successfully followed up are presented in Table 1. Age, waist circumference, body mass index, blood pressure, and serum levels of total cholesterol，triglycerides, low-density lipoprotein cholesterol, aspartate aminotransferase, alanine aminotransferase, glutamyltranspeptidase, fasting blood glucose, and uric acid all tended to decrease with higher ApoA1 levels (all P＜0.01）.

**Table 1**. Relationship between serum apolipoprotein A1 level and metabolic syndrome and related indexes

| Varialble | Overall  N=12823 | serum ApoA1 levels（g/L） | | | | F value | p value |
| --- | --- | --- | --- | --- | --- | --- | --- |
|  |  | Q1  （≤1.18） | Q2（1.19~1.32） | Q3（1.33~1.55） | Q4  （＞1.56） |  |  |
| Age(year） | 47.2±15.1 | 44.5±14.5 | 45.2±14.9 | 47.1±15.5 | 51.7±14.4 | 146.977 | <0.001 |
| waist circumference(cm） | 82.7±9.1 | 85.8±8.4 | 83.8±8.8 | 81.7±8.9 | 79.4±8.9 | 314.529 | <0.001 |
| body mass index（kg/m^2^） | 23.5±3.1 | 24.4±3.0 | 23.8±3.1 | 23.2±3.0 | 22.6±3.1 | 204.758 | <0.001 |
| systolic blood pressure（mmHg） | 122.9±16.1 | 122.8±15.1 | 122.7±15.7 | 122.3±16.3 | 123.9±17.2 | 6.027 | <0.001 |
| diastolic blood pressure（mmHg） | 78.1±10.1 | 78.3±9.8 | 78.1±10.1 | 77.6±10.5 | 78.5±10.5 | 4.824 | 0.002 |
| total cholesterol（mmol/L) | 4.8(4.2,5.4) | 4.5(3.9,5.1) | 4.7(4.2,5.3） | 4.8(4.5,5.4) | 5.2(4.6,5.8) | 178.674^*^ | <0.001 |
| triacylglycerol (mmol/L) | 1.2(0.8,1.7) | 1.4(1.0,2.1) | 1.2(0.8,1.7) | 1.1(0.8,1.6) | 1.0(0.7,1.4) | 513.266^*^ | <0.001 |
| LDL cholesterol (mmol/L) | 2.7±0.7 | 2.6±0.7 | 2.7±0.7 | 2.7±0.7 | 2.7±0.8 | 25.616 | <0.001 |
| aspartate aminotransferase (U/L) | 21(18,26) | 21(18,26) | 21(17,26) | 21(18,25) | 22(19,27) | 72.281^*^ | <0.001 |
| alanine aminotransferase (U/L) | 19(13,27) | 21(14,31) | 19(14,29) | 18(13,26) | 17(13,24) | 220.690^*^ | <0.001 |
| glutamyltranspeptidase (U/L) | 22(16,33) | 24(17,35) | 22(16,33) | 21(15,32) | 20(15,31) | 137.637^*^ | <0.001 |
| fasting plasma glucose（mmol/L) | 5.2±0.9 | 5.2±1.0 | 5.2±0.9 | 5.2±0.9 | 5.3±1.1 | 18.151 | <0.001 |
| serum uric acid (μmol/L） | 325.1±82.3 | 350.0±79.3 | 334.9±78.8 | 320.7±81.1 | 294.2±79.3 | 287.738 | <0.001 |
| glycosylated hemoglobin (%) | 5.1(4.8,5.5) | 5.1(4.7,5.5) | 5.1(4.8,5.5) | 5.1(4.8,5.5) | 5.1(4.8,5.4) | 0.411* | 0.938 |

Data are expressed as mean (SD) or median (IQR). * Z value; LDL, low density lipoprotein;

**Comparison of clinical and laboratory indexes between NAFLD and normal subjects**

Subjects with NAFLD had higher age,waist circumference, body mass index, systolic and diastolic blood pressure, total cholesterol, triacylglycerol, low density lipoprotein cholesterol,alanine aminotransferase, aspartate aminotransferase, glutamyltranspeptidase, fasting plasma glucose, serum uric acid levels, serum glycosylated hemoglobin level, and a lower Apolipoprotein A1 level than subjects without NAFLD. As noticed in Table 2.

**Table 2.** Comparison of clinical and laboratory indexes between NAFLD and normal subjects

| Varialble | | NAFLD absent（n=9676） | NAFLD resent（n=3147） | T value | | p value |
| --- | --- | --- | --- | --- | --- | --- |
| sex(male/female) | 6038/3638 | | 2411/706 | 243.771# | <0.001 | |
| age(year） | 46.1±15.4 | | 49.7±13.8 | 10.817 | <0.001 | |
| waist circumference（cm） | 80.3±8.2 | | 90.1±7.6 | 58.917 | <0.001 | |
| body mass index（kg/m^2^） | 22.6±2.7 | | 26.3±2.8 | 64.971 | <0.001 | |
| systolic blood pressure（mmHg） | 120.7±15.6 | | 129.7±15.5 | 28.089 | <0.001 | |
| diastolic blood pressure（mmHg） | 76.7±9.7 | | 82.5±10.2 | 28.814 | <0.001 | |
| total cholesterol（mmol/L) | 4.7(4.2,5.3) | | 5.1(4.5,5.7） | 8.317* | <0.001 | |
| triacylglycerol (mmol/L) | 1.0(0.8,1.4) | | 1.7(1.3,2.5) | 20.643* | <0.001 | |
| LDL cholesterol (mmol/L) | 2.6±0.7 | | 2.9±0.8 | 15.903 | <0.001 | |
| aspartate aminotransferase (U/L) | 20(17,24) | | 25(20,31) | 12.794* | <0.001 | |
| alanine aminotransferase (U/L) | 16(12,23) | | 29(20,43) | 20.198* | <0.001 | |
| glutamyltranspeptidase (U/L) | 19(15,28) | | 34(24,51) | 20.366 | <0.001 | |
| fasting plasma glucose（mmol/L) | 5.1±0.9 | | 5.5±1.2 | 18.508 | <0.001 | |
| serum uric acid (μmol/L） | 311.0±77.5 | | 368.5±81.4 | 35.692 | <0.001 | |
| glycosylated hemoglobin (%) | 5.1(4.8,5.4) | | 5.2(4.9,5.6) | 4.815* | <0.001 | |
| Apolipoprotein A1 (g/L) | 1.4±0.3 | | 1.3±0.3 | 16.028 | <0.001 | |

Data are expressed as mean (SD) or median (IQR). # χ 2 value; * Z value; LDL, low density lipoprotein;
